# Prognosis of Vital Teeth Involved in Large Cystic Lesions After a Surgical Intervention: A Longitudinal Ambidirectional Cohort Study

**DOI:** 10.3390/dj13020083

**Published:** 2025-02-15

**Authors:** Khalid A. Merdad, Maha Shawky, Khalid A. Aljohani, Rawia Alghamdi, Saja Alzahrani, Omar R. Alkhattab, Abdulaziz Bakhsh

**Affiliations:** 1 Endodontic Department, Faculty of Dentistry, King Abdulaziz University, Jeddah 22254, Saudi Arabia; oralkhattab@kau.edu.sa; 2 Oral and Maxillofacial Surgery Department, Cairo University, Cairo 12613, Egypt; maha.shawky@dentistry.cu.edu.eg; 3 Oral Diagnostic Sciences Department, Faculty of Dentistry, King Abdulaziz University, Jeddah 22254, Saudi Arabia; koalgehani@kau.edu.sa; 4 Dental Intern, Faculty of Dentistry, King Abdulaziz University, Jeddah 22254, Saudi Arabia; ralghamdi0592@stu.kau.edu.sa (R.A.); salzahrani0887@stu.kau.edu.sa (S.A.); 5 Restorative Department, Faculty of Dental Medicine, Umm Al-Qura University, Makkah 24381, Saudi Arabia; aabakhsh@uqu.edu.sa

**Keywords:** odontogenic cyst, endodontic treatment, teeth vitality, prognosis

## Abstract

**Background/Objectives:** Large cystic lesions in the maxillofacial region present a challenge for clinicians due to their impact on the health and functionality of the involved teeth. This longitudinal, ambidirectional cohort study aimed to evaluate the prognosis of vital teeth affected by large cystic lesions following surgical intervention. **Methods**: Data were gathered from patients at King Abdulaziz University Dental Hospital and King Fahad General Hospital in Jeddah, Saudi Arabia, between July 2021 and August 2022. Seventeen individuals with large jawbone cysts were included in the study. Clinical and radiographic assessments were performed including sensibility testing and the function of teeth. The results indicated a linear relationship between the size of postoperative bony defects and the sensibility testing of teeth. **Results:** Of the 63 examined teeth, 54% did not receive endodontic treatment, 33% had pre-surgical treatment, and 12.7% had post-surgical treatment. The study revealed a high prevalence of inflammatory cysts, particularly in the maxilla. Surgical enucleation was the primary treatment modality, with few postoperative complications. **Conclusions:** This study highlights the necessity for standardized follow-up protocols and more extensive research to develop universal guidelines for managing large cystic lesions affecting vital teeth. Understanding the prognosis of cystic lesions is crucial for effective treatment planning and ensuring optimal patient outcomes.

## 1. Introduction

Radiographic pathologies of the maxilla or mandible can present as radiolucent, radiopaque, or mixed and may be associated with adjacent vital or non-vital teeth. They are differentially diagnosed as granulomas, abscesses, cysts, and benign or malignant tumors [1,2]. Histological assessment of the lesion following its enucleation can confirm the actual diagnosis.

It was well established by Kramer [3] that cysts are defined as a pathological cavity filled with fluid, semifluid, or gaseous contents that are not associated with pus. They are mostly lined with epithelium, except for three different cystic lesions when referring to the maxillofacial region, namely, mucous extravasation cysts of the salivary glands, aneurysmal bone cysts, and solitary bone cysts [4]. These cysts are also referred to as “pseudo-cysts” or “cavity” cysts [5].

In 1992, the World Health Organization (WHO) classified odontogenic cysts into two main categories: developmental and inflammatory cysts. However, recent updates by the WHO preferred to discuss cysts of the jaw without subdivision. Cysts of the jaw include several types, including inflammatory odontogenic cysts, which include the radicular cyst, inflammatory collateral cysts, and the developmental odontogenic cysts, which include the gingival cysts, calcifying odontogenic cysts, glandular odontogenic cysts, and odontogenic keratocytes [6].

Odontogenic inflammatory cysts are among the most common cystic lesions in the human jaw, with prevalence ranging from 52% to 68% and with a male predominance. The most common type of odontogenic inflammatory cyst is the radicular cyst, followed by the dentigerous cyst [7].

When treating cysts, an assessment of tooth restorability must be carried out first and then a decision should be made about whether to extract or perform nonsurgical endodontic therapy. A combination of endodontic nonsurgical treatment, enucleation, marsupialization, decompression, or fenestration is preferred to treat cystic lesions. Recent case reports and series found that a custom-made removable appliance can be used for the decompression of the cystic lesion, which consequently reduces the risk of complications associated with the surgical treatment [8,9]. Consequently, follow-up is recommended for one year or two years [10,11,12]. Histopathologically, the characteristics of inflammatory cysts includes the stratified squamous epithelium, which lines the cystic cavity. It may also exhibit exocytosis, spongiosis, or hyperplasia [13,14].

The potential risk of morbidity arises in the surgical removal of large cysts involving sound vital teeth [15]. Therefore, they must be endodontically treated before or after surgical enucleation. If the radiolucency is not resolved in the follow-up visit for lesions smaller than 2 mm in diameter, the management is nonsurgical endodontic retreatment. On the other hand, if the cyst initially exceeds 2 mm, the tooth is not suitable for conventional endodontics, or the lesion increases in size or does not reduce during the observation period, then periapical surgery should be performed, followed by a biopsy to rule out other oral pathologies. Furthermore, when a paradental cyst is associated with first or second molars, the treatment will include the enucleation of the lesion to preserve the involved tooth. However, if it is related to third molars, the treatment is the extraction of the offending tooth with surgical excision of the lesion [12,13].

There is no clear consensus in the literature regarding the prognosis of the involved teeth, which raises the question of how vital teeth involved in large cysts sustain their vitality after the vascularity has been compromised due to surgical intervention. Most of the previous studies are case reports [16,17,18,19,20,21], and there is little published data on the proper guidelines or substantial studies to support them.

Therefore, the aim of this study was to clinically examine the condition of the involved teeth in follow-up visits after the surgical removal of large cysts to assess their prognosis in terms of loss of sensibility and function.

## 2. Materials and Methods

### 2.1. Study Design

This cohort study included a retrospective, and a prospective phase carried out from July 2021 to August 2022. Data from patients with large jawbone cysts seen in King Abdulaziz University Dental Hospital (KAUDH) and King Fahad General Hospital (KFGH) in Jeddah, Saudi Arabia, were collected. The follow-up records were obtained from the maxillofacial surgery departments.

### 2.2. Ethical Approval

The study was approved by the Research Ethics Committee at the Faculty of Dentistry, King AbdulAziz University (Protocol No. 329-11-21). Informed written consent was signed by patients after explaining the study aims. The study was conducted in accordance with the Declaration of Helsinki.

### 2.3. Sample Selection

The inclusion criteria involved any patient with a large cyst (>2 cm) involving two or more teeth. However, patients under 18-years old, with any bone diseases, and patients with cysts < 2 cm in size or who had had any previous treatment for the same lesion, with non-vital teeth before the surgery or patients who had had their involved teeth extracted, or who had any acute or acute exacerbation of a chronic condition, on any medication that may affect bone metabolism or may interfere with the results of the study were excluded from the study. Also, any eligible patient who was lost to follow-up or refused to give informed consent or was not indicated for surgical treatment was excluded from the study.

**Figure 1:**
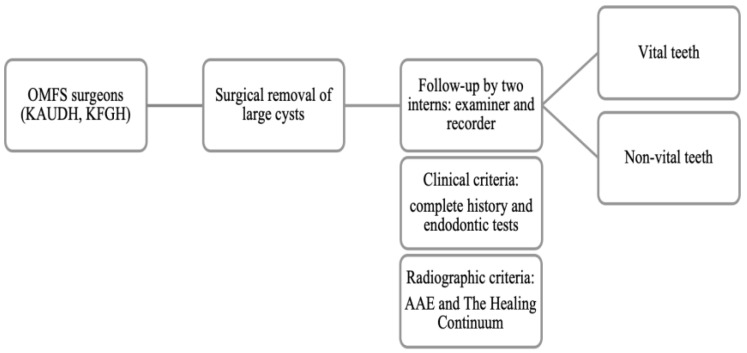
Flow chart of the follow-up process. (OMFS: Oral and Maxillofacial Surgeron).

In the retrospective phase, two investigators obtained a list of patients (*n* = 76) who were diagnosed with cysts in both of the KAUDH and KFGH centers. Electronic preoperative medical records were thoroughly checked, and data were collected and categorized in a self-structured table sheet for each patient. The collected data included age, sex, complete history including medical and dental records, smoking habits, history of symptoms, and teeth involved in the lesion, as well as the endodontic diagnosis. Additional information included the type of surgical intervention (enucleation, marsupialization, or decompression) with timing and complications, and the histopathological diagnosis. Pre- and post-surgical radiographs and follow-up periods were also obtained (Figure 1).

### 2.4. Study Variables

In the prospective phase, all patients were recalled up to 36 months following surgery. A thorough clinical and radiographic examination was carried out according to a preplanned checklist, which included extra- and intraoral examination, sensibility testing, the presence of any postoperative deformity, and radiographic examination including periapical radiograph (PA) (Kodak RVG5200: Carestream Health, Rochester, NY, USA), and limited field of view (40 mm^3^) cone-beam computed tomography (CBCT)—Op 3D Pro (Kavo, Charlotte, NC, USA); 90 kV, 5.0 mA, and 17.5 s—to assess the surgical outcome and prognosis of the involved teeth during the follow-up intervals.

Information derived from CBCT included the size of the bony defect, its site, whether the involved teeth were endodontically treated, and bony defect obliteration (complete or incomplete). The radiographic criteria to diagnose the outcome of involved teeth were determined according to the American Association of Endodontics and The Healing Continuum criteria (healed, healing, disease, functional retention) (Table 1) [22].

### 2.5. Data Collection During Recall Visit

First, the chief complaint, the medical, dental, and current history of symptoms and the post-surgical complications were updated. Second, clinical examinations were carried out, beginning with extra- and intraoral screening, and then assessing the involved teeth using a cold sensibility test with dichlorodifluoromethane gas (−50 °C, Endo-frost). The response was recorded as normal (N), mild (+), moderate (++), lingering pain (L), and no response (0).

Moreover, a basic periodontal examination was conducted as well as percussion, palpation, and mobility tests. In addition, the radiographic changes were assessed using CBCT scans. Assessments of the CBCT scans were performed by two calibrated clinicians. The images were viewed using Planmeca Romexis software V4.5.0.R (Planmeca Oy Inc., Helsinki, Finland, 2020). The following parameters were analyzed: postoperative volumic dimensions of the bony defect (height, width, depth), site (anterior/posterior, maxilla/mandible), whether the bone healing was proceeding or not, whether obliteration of the bony defect was complete or partial/incomplete, and the quality of the root canal treatment if teeth were involved.

### 2.6. Statistical Analysis

This ambidirectional cohort study evaluated the associations and correlations between the independent variable (the surgical removal of large cystic lesion) and the dichotomous dependent variable (the sensibility testing of the involved teeth). Descriptive statistics were determined for the study variables using IBM SPSS Statistics Ver. 21.0 software.

## 3. Results

**Figure 2:**
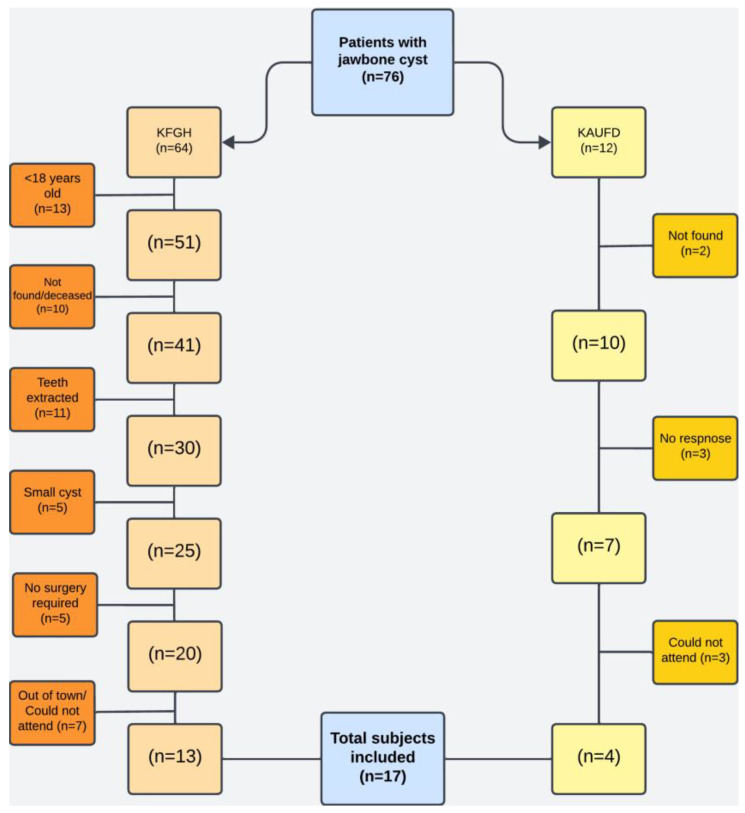
Flowchart of the sample selection of patients for follow-up.

Of the 76 patients (n = 64 KFGH; n = 12 KAUFD), 12 participants were <18 years old and were excluded. Eleven patients had their involved teeth extracted, and five had lesions < 2 cm. Furthermore, 25 patients were not reachable or could not attend the recall appointment, while five were not indicated for surgical treatment. Finally, 17 patients with 63 teeth were included in the study (Figure 2).

Of the 17 patients included in the study, 64.7% (n = 11) were males and 35.3% (n = 6) were females. The mean age was 35.88 ± 9.44 (range 20–56) years old. Most of the study participants were free from any other medical condition (82.4%, n = 14), and 17.6% (n = 3) reported a history of hypothyroidism, hypertension, or asthma. Finally, 29.4% (n = 5) were smokers (Table 2).

All cysts underwent surgical enucleation. A total of 70.6% (n = 12) cysts were found in the maxilla compared to 29.4% (n = 5) in the mandible. In addition, lesions in the right quadrant were found in 23.5% (n = 4) of the samples, while 32.5% (n = 6) of the lesions were in the left quadrant. Meanwhile, lesions crossing the midline were found in 41.2% of the sample (n = 7) (Table 3).

The cysts were mainly found higher in the anterior region than in the posterior (47.1% (n = 8); 17.6% (n = 3), respectively). Preoperative swelling was the most common symptom, reported by 70.6% (n = 12) participants, followed by pain (52.9%, n = 9). The histopathological diagnosis differed for each patient, with the most common type being radicular cysts (53.8%, n = 7), followed by dentigerous cysts (23%, n = 3), and orthokeratinized odontogenic cysts (15.4%, n = 2). The number of teeth involved in these large cysts ranged between two and eight, and 47.1% (n = 8) of these cysts involved four teeth or more. Buccal/labial or lingual/palatal bone perforations were seen in 58.8% (n = 10) of the samples, while other effects on neighboring structures included expansion (41.2%, n = 7) and displacement (41.2%, n = 7) (Table 3).

Regarding quality of life after surgery, 23.5% (n = 4) of the patients reported that they were unable to function when using their involved teeth. While 41.2% (n = 7) of patients stated problems regarding esthetics, none of the patients reported any deformity or asymmetry in the face (Table 3).

Less than one-fifth (17.6%, n = 3) of the patients reported no previous follow-up visit, whereas 35.3% (n = 6) visited the hospital only one week after the surgery. At the time of their prospective follow-up visit, 47.1% (n = 8) of the patients had had their cyst removed about three to six months earlier, while the maximum follow-up period was 32 months (Table 3).

Of the 63 teeth examined, three were tender to percussion (4.8%) and only one was tender to palpation (1.6%). In addition, 61.9% (n = 39) of the teeth had no mobility, while the remaining teeth (n = 24) had grade 1 mobility based on Miller’s index [23]. Measurements of the probing depth were divided into ≤4 (88.9%, n = 56) and >4 mm (11.1%, n = 7). When assessing non-root-canal-treated teeth’s response to sensibility using the cold test, 46% (n = 29) had no response, while 39.7% (n = 25) responded similarly to the control tooth. In the pulpal and periapical diagnosis and according to the American Association of Endodontics, 42.9% (n = 27) of the teeth were previously treated for asymptomatic apical periodontitis, followed by normal pulp with asymptomatic apical periodontitis at 28.6% (n = 18), and normal pulp with normal apical tissue at 9.5% (n = 6). Most of the teeth were healing according to The Healing Continuum [22], and those showing symptoms only accounted for 4.8% (n = 3) of the teeth. Of all involved teeth, 54% (n = 34) did not receive any endodontic treatment. On the other hand, 33% (n = 21) had pre-surgical endodontic treatment, and 12.7% (n = 8) had root canal treatment carried out after cystic surgery (Table 4).

**Figure 3:**
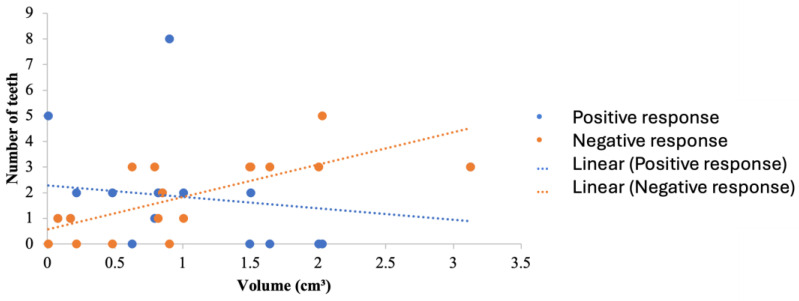
The association between the size of the bony defect following surgical removal of the cyst and the response of the involved teeth.

**Figure 4:**
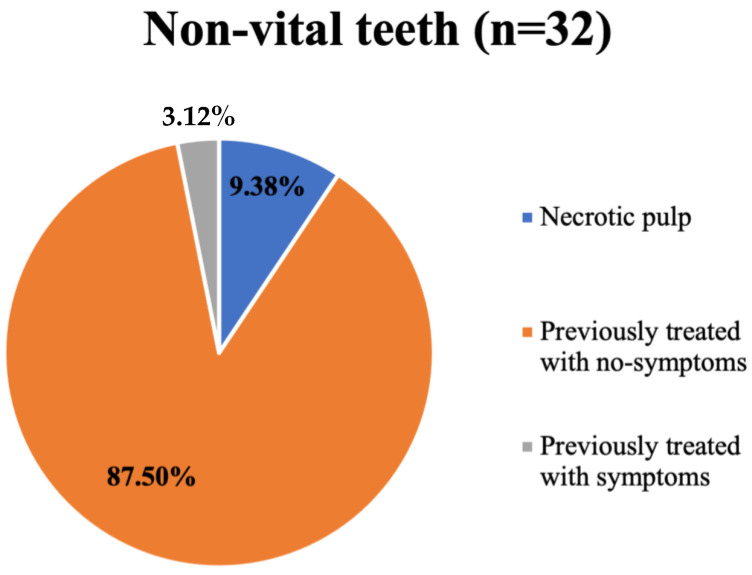
A pie chart illustrating the number of non-vital teeth involved in the study.

These results revealed a linear association between the size of the postoperative bony defect and the sensibility testing of the involved teeth (Figure 3). Of the 32 non-vital teeth diagnosed in the follow-up appointment, 87.5% were previously treated and asymptomatic. Moreover, 9.38% were necrotic teeth, and 3.12% were previously treated and had symptoms (Figure 4).

## 4. Discussion

Cystic lesions are hard to differentiate from periapical granuloma radiographically [24,25]. Histopathologic examination is the only reliable method for differentiating between these lesions [26]. Furthermore, surgical enucleation of the cystic lesions may disturb the blood flow within the teeth associated with the lesion. Therefore, the fate of the vitality of teeth involved in cystic lesions is unknown. Several factors could play a role in the vitality of teeth involved in odontogenic cysts such as caries or trauma [27]. In this study, there was no association between smoking or medical conditions (hypertension, hypothyroidism, asthma) and the prognosis of the involved teeth after the surgical removal of cysts. Such cases displayed no symptoms and, therefore, were not considered for root canal treatment.

The histological analysis of cysts associated in this study revealed that the radicular cyst, which is caused by inflammation, exhibited the largest number (53.8%). The results are in alignment with the study conducted by Jones, Craig, and Franklin [7] who reported that inflammatory cysts including radicular cysts accounted for 66% of the samples compared to 31.1% of developmental cysts [7]. Cysts of the jaw caused by inflammation require a source of inflammation or infection to develop and grow. In the present study, almost half of the teeth did not respond to sensibility testing, which could be the source of infection causing the inflammatory cysts. Moreover, most of the cystic lesions detected in this study were located in the maxillary arch (70.6%), and this conforms with previous studies reporting that the maxilla is the most commonly affected site [11,28,29].

Treating large cysts can be achieved using different techniques such as marsuplization, decompression, and enucleation [30]. In this study, the treatment modality chosen by the practitioners was enucleation. Thus, no comparison could be made between different surgical treatment types. Many studies reported that the best treatment option for large odontogenic cysts is performing both surgical treatment and root canal treatment [18,20,21,22]. Postoperatively, only one patient complained of temporary paresthesia at the surgical site; this could be due to the proximity of the cystic lesion to the anterior or/and middle superior alveolar nerve, as the cyst extended from the left central incisor to the left first premolar. Two other patients complained of discomfort at the surgical site.

Recently, Yi, Bing and Zhao [15] reported that there was no agreement regarding the extraction of the affected tooth in cases of root involvement in odontogenic keratocysts. They added that there is controversy around whether root canal treatment should be necessary before or after the enucleation of the cystic lesions [15]. On the other hand, Al-Huwaizi H. and Issa SA. [31], in their case report, successfully proved that the healing of large cysts could be achieved by combining surgical enucleation and nonsurgical root canal treatment [31].

In the literature, it was found that larger lesions (>5 mm) can be associated with delayed bone healing [32,33]. In this study, 10 patients had cysts perforating either one or both bone plates, and this justifies the delay in bone healing. Furthermore, large lesions perforating the bone plates may heal with a fibrous scar and present as a radiolucent cavity in the radiographs, as reported by Santamaría, et al. [34] and Chiapasco, et al. [35], respectively; this could lead to the misinterpretation of the radiographic analysis.

The dimensions of the cysts, as measured by ellipsoid volume, ranged between 0.008 and 3.126 cm^3^. In this study, it was found that the larger the bony defect, the more the response to sensibility testing may be compromised. Four out of seventeen patients retained the vitality of all of their involved teeth; however, it can be noted that all of their bony defects were less than 1 cm^3^ in size. However, the teeth involved in cystic lesions in five patients did lose their response to sensibility testing; the cavity size was greater than 1 cm^3^ in four out of five patients. Mosquera-Barreiro et al. [36] found that there is a constant relation between age and lesion size, reporting that the older the patient, the larger the lesion would be [36]. In this study, it was shown that young patients had the smallest defect size (20 years old), had the longest follow-up period (32 months), and all teeth involved in the cystic lesion retained their vitality.

In the current study, three teeth were considered to have a necrotic pulp as they did not respond to sensibility testing, which accounted for 9.4% out of all non-vital teeth. In addition, one patient had four involved teeth, all diagnosed prior to surgery, two of which were non-vital and endodontically treated. One of these two teeth turned out to be symptomatic six months postoperatively. In those cases, root canal treatment was a necessity for the three necrotic teeth, and retreatment was conducted for the other symptomatic tooth. However, the remaining teeth remained responsive to sensibility testing, and patients were asymptomatic.

The European Society of Endodontology recommended the use of CBCT prior to surgical treatment [37]. However, as a limitation of the study, baseline CBCT scans were not taken for patients prior to surgery, except for five; therefore, accurate measurements of the lesion size, proximity to vital structures, and the extent of involved teeth could not be made accurately from PA alone. Moreover, six patients who had records of previous follow-up visits were missing, and the inconsistent follow-up intervals between subjects was also a limitation. However, this calls for a standardized regimen. Finally, more studies with longer follow-up periods and larger sample sizes are needed in order to develop a universal guideline for pre- and post-surgical protocols for the follow-up of all cases, including sound teeth involved in cystic lesions.

## 5. Conclusions

Although the prognosis for vital teeth affected by extensive cystic lesions is compromised due to the inadequate blood supply postoperatively, surgical excision of the cyst is recommended. Furthermore, with adequate follow-up, not all teeth included in the cyst require preoperative root canal treatment. Additionally, the teeth involved in the cyst should be monitored to assess for the development of symptoms. In such cases, postoperative treatment is necessary. Finally, a standardized protocol should be established to ensure consistent follow-up intervals for teeth within the cystic lesion.

This study is limited by the heterogeneity of the sample and the position of the treated teeth, which may introduce bias. Additionally, the small sample size limits the generalizability of the findings.

## Figures and Tables

**Table 1:** The Healing Continuum according to Friedman and Mor [22].

Term	Clinical Findings	Radiographic Findings
Healed	Clinical normalcy (Asymptomatic)	Radiographic normalcy (No RL)
Healing	Asymptomatic	RL reduced
Disease	Symptomatic or Asymptomatic	No RL RL unchanged RL enlarged RL emerged
Functional Retention	Asymptomatic	Not considered

RL= radiolucency.

**Table 2:** Demographic characteristics of the study participants (n = 17 subjects).

Age	Mean ± SD	35.88 ± 9.44
Gender	Female	6 (35.3%)
	Male	11 (64.7%)
Medical history	Healthy	14 (82.4%)
	Medical condition	3 (17.6%)
Smoking	Smoker	5 (29.4%)
	Non-smoker	12 (70.6%)

**Table 3:** Characteristics of the cysts and the follow-up visits (n = 17).

		n (%)
Arch	Maxilla	12 (70.6)
	Mandible	5 (29.4)
Side	Right	4 (23.5)
	Left	6 (35.3)
	Crossing the midline	7 (41.2)
Location	Anterior area	8 (47.1)
	Posterior area	3 (17.6)
	Both	6 (35.3)
Pain	Yes	9 (52.9)
	No	8 (47.1)
Swelling	Yes	12 (70.6)
	No	5 (29.4)
Abscess	Yes	4 (23.5)
	No	13 (76.5)
Dimension (cm^3^)	Mean ± SD	1.0 ± 0.8
	<1 cm^3^	10 (58.8)
	≥1 cm^3^	7 (41.2)
Relation to bony structures (Expansion)	Yes	7 (41.2)
	No	10 (58.8)
Relation to neighboring structures (Displacement)	Yes	7 (41.2)
	No	10 (58.8)
Relation to bony cortices (Perforation)	Yes	10 (58.8)
	No	7 (41.2)
Number of teeth involved	2 teeth	4 (23.5)
	3 teeth	5 (29.4)
	≥4 teeth	8 (47.1)
Patient functions	Unable to function	4 (23.5)
	Functioning	13 (76.5)
Facial asymmetry	Symmetric	17 (100)
	Asymmetric	0
Esthetic problems	Yes	7 (41.2)
	No	10 (58.8)
Deformity	Yes	0
	No	17 (100)
Histopathological diagnosis	Radicular cysts	7 (53.8)
	Dentigerous cysts	3 (23)
	Orthokeratinized odontogenic cysts	2 (15.4)
	Sebaceous cysts	1 (7.7)
Surgical procedure	Enucleation	17 (100)
Postoperative complications	Yes	3 (17.6)
	No	14 (82.4)
Type of complication	Temporary parasthesia	1 (33.3)
	Discomfort at surgical site	2 (66.6)
Follow-up visits in weeks	None	3 (17.6)
	1 week	6 (35.3)
	2 weeks	5 (29.4)
	≥3 weeks	3 (17.6)
Current follow-up visits in months	3–6 months	8 (47.1)
	7–12 months	5 (29.4)
	13–36 months	4 (23.5)

**Table 4:** Current clinical examination of the involved teeth (n = 63).

		n (%)
Percussion	Normal	60 (95.2)
	Abnormal	3 (4.8)
Palpation	Normal	62 (98.4)
	Abnormal	1 (1.6)
Mobility	Normal	39 (61.9)
	Grade 1	24 (38.1)
	Grade 2	0
	Grade 3	0
Probing	≤4 mm	56 (88.9)
	>4 mm	7 (11.1)
Cold test	Normal	25 (39.7)
	Delayed	2 (3.3)
	Positive (mild pain)	5 (7.9)
	Positive (moderate pain)	0
	Lingering	2 (3.2)
	No response	29 (46.0)
Diagnosis (AAE)	Normal pulp with normal apical tissue	6 (9.5)
	Normal pulp with asymptomatic apical periodontitis	18 (28.6)
	Normal pulp with symptomatic apical periodontitis	1 (1.6)
	Reversible pulpitis with asymptomatic apical periodontitis	3 (4.8)
	Reversible pulpitis with symptomatic apical periodontitis	1 (1.6)
	Symptomatic irreversible pulpitis with asymptomatic apical periodontitis	2 (3.2)
	Necrotic pulp with asymptomatic apical periodontitis	3 (4.8)
	Previously treated with normal apical tissue	1 (1.6)
	Previously treated with asymptomatic apical periodontitis	27 (42.9)
	Previously treated with symptomatic apical periodontitis	1 (1.6)
Outcome (The Healing Continuum)	Healing	60 (95.2)
	Disease	3 (4.8)
Endodontic treatment	None	34 (54.0)
	Preoperative	21 (33.3)
	Postoperative	8 (12.7)

## Data Availability

The research data are available from the corresponding author.
